# Regulation of mitochondrial network architecture and function in mesenchymal stem cells by micropatterned surfaces

**DOI:** 10.1093/rb/rbae052

**Published:** 2024-05-07

**Authors:** Zixuan Dong, Weiju Han, Panyu Jiang, Lijing Hao, Xiaoling Fu

**Affiliations:** The Second Affiliated Hospital, School of Biomedical Sciences and Engineering, South China University of Technology, Guangzhou 511442, China; National Engineering Research Center for Tissue Restoration and Reconstruction and Innovation Center for Tissue Restoration and Reconstruction, Guangzhou 510006, China; Laboratory of Biomedical Engineering of Guangdong Province, South China University of Technology, Guangzhou 510006, China; National Engineering Research Center for Tissue Restoration and Reconstruction and Innovation Center for Tissue Restoration and Reconstruction, Guangzhou 510006, China; Laboratory of Biomedical Engineering of Guangdong Province, South China University of Technology, Guangzhou 510006, China; School of Materials Science and Engineering, South China University of Technology, Guangzhou 510006, China; The Second Affiliated Hospital, School of Biomedical Sciences and Engineering, South China University of Technology, Guangzhou 511442, China; National Engineering Research Center for Tissue Restoration and Reconstruction and Innovation Center for Tissue Restoration and Reconstruction, Guangzhou 510006, China; Laboratory of Biomedical Engineering of Guangdong Province, South China University of Technology, Guangzhou 510006, China; National Engineering Research Center for Tissue Restoration and Reconstruction and Innovation Center for Tissue Restoration and Reconstruction, Guangzhou 510006, China; Laboratory of Biomedical Engineering of Guangdong Province, South China University of Technology, Guangzhou 510006, China; School of Materials Science and Engineering, South China University of Technology, Guangzhou 510006, China; National Engineering Research Center for Tissue Restoration and Reconstruction and Innovation Center for Tissue Restoration and Reconstruction, Guangzhou 510006, China; Laboratory of Biomedical Engineering of Guangdong Province, South China University of Technology, Guangzhou 510006, China

**Keywords:** micropatterned surface, mesenchymal stem cells, mitochondrial network architecture, mitochondrial function, regulation of mitochondria

## Abstract

Mitochondrial network architecture, which is closely related to mitochondrial function, is mechanically sensitive and regulated by multiple stimuli. However, the effects of microtopographic cues on mitochondria remain poorly defined. Herein, polycaprolactone (PCL) surfaces were used as models to investigate how micropatterns regulate mitochondrial network architecture and function in rat adipose-derived stem cells (rASCs). It was found that large pit (LP)-induced rASCs to form larger and more complex mitochondrial networks. Consistently, the expression of key genes related to mitochondrial dynamics revealed that mitochondrial fusion (MFN1 and MFN2) and midzone fission (DRP1 and MFF) were increased in rASCs on LP. In contrast, the middle pit (MP)-enhanced mitochondrial biogenesis, as evidenced by the larger mitochondrial area and higher expression of PGC-1. Both LP and MP promoted ATP production in rASCs. It is likely that LP increased ATP levels through modulating mitochondrial network architecture while MP stimulated mitochondria biogenesis to do so. Our study clarified the regulation of micropatterned surfaces on mitochondria, highlighting the potential of LP and MP as a simple platform to stimulate mitochondria and the subsequent cellular function of MSCs.

## Graphical Abstract

**Figure:**
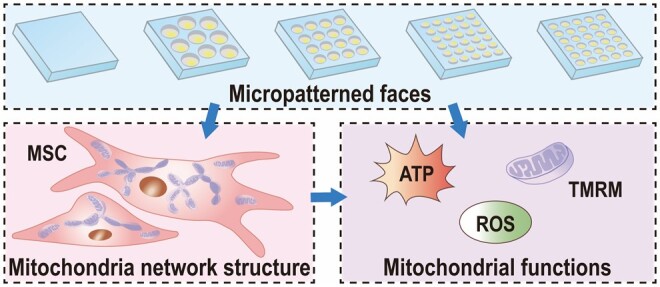

## Introduction

Mitochondria are the primary energy-producing organelles of eukaryotic cells [1, 2]. In addition to producing ATP, they also participate in apoptosis, cell senescence, intermediate metabolism, calcium signal transmission, and other cellular processes [1, 3, 4]. Mammalian cells can have up to a few thousand mitochondria, depending on the type of cell and the metabolic state [5]. In cells, the mitochondria exist as a large tubular complex network extending throughout the cytosol and in close contact with other organelles (e.g. nucleus, endoplasmic reticulum and Golgi apparatus) and the cytoskeleton. The architecture of the mitochondrial network is highly dynamic, shifting frequently between a fragmented state and a tubular continuum through fission and fusion of the outer and inner membranes [6]. Studies to date have demonstrated that the dynamic mitochondrial network is closely related to mitochondrial function [7]. For example, it has been reported that changes in mitochondrial architecture serve as a bioenergetic adaptation mechanism to metabolic demands [8]. When there is a strong need for energy, mitochondria tend to be elongated in shape with developed cristae, but when there is a low demand for energy, mitochondria tend to be small, fragmented and spherical with underdeveloped cristae [9]. In addition, fission of the mitochondrial network occurs in reaction to injury or cancer [10–12].

Mesenchymal stem cells (MSCs) have been widely used in regenerative medicine, but it is still a challenge to boost their therapeutic functions via simple approaches. Recent studies have shown that mitochondria are critical for the function and fate of stem cells [13, 14]. During growth, migration, differentiation, aging and even death, MSCs change their mitochondrial architecture in a particular way [15]. For example, the formation of the mitochondrial crest occurs concurrently with osteogenic differentiation of bone marrow-derived MSCs [16]. Additionally, mitochondria are uniformly distributed throughout the cytoplasm of differentiated MSCs, while they are primarily clustered around the nucleus in undifferentiated MSCs [16]. During differentiation, the ratio of mitochondrial to cytoplasmic area increased concurrently [17]. Correspondingly, the levels of mitochondrial DNA copies, respiratory enzyme proteins, oxygen consumption rate, genes associated with mitochondrial biogenesis, and intracellular ATP content all increase when MSCs undergo differentiation [18]. Thus, it is possible to enhance the function of MSCs by modulating their mitochondria.

Mitochondria are mechanically sensitive organelles. They are closely bound to the cytoskeleton, thereby being capable of sensing external forces through the deformation of the cytoskeleton [19]. Some pioneering studies have shown that external mechanical stimulation can regulate mitochondrial dynamics, biogenesis, and mitochondrial autophagy through the cytoskeleton [20–25]. One typical example is that mitochondria in cells exposed to repetitive stretch cycles tend to fuse into larger or more complicated networks together with increased membrane potential and higher ATP production [26, 27]. These findings highlight the potential of modulating mitochondrial architecture and function by external mechanical forces. As one key physical characteristic of substrates, microtopography has been well recognized as a crucial regulator of a variety of cell functions, such as morphological alterations, differentiation and paracrine function [28, 29]. However, the effects of surface microtopography on mitochondria remain poorly defined. Considering that microtopography can change cell morphologies and the internal cytoskeleton as well as other mechanical stresses, we hypothesize that surface microtopographies may modulate mitochondrial architecture and function in MSCs and that certain microtopographies may induce higher mitochondrial activity and ATP production.

**Figure 1.**
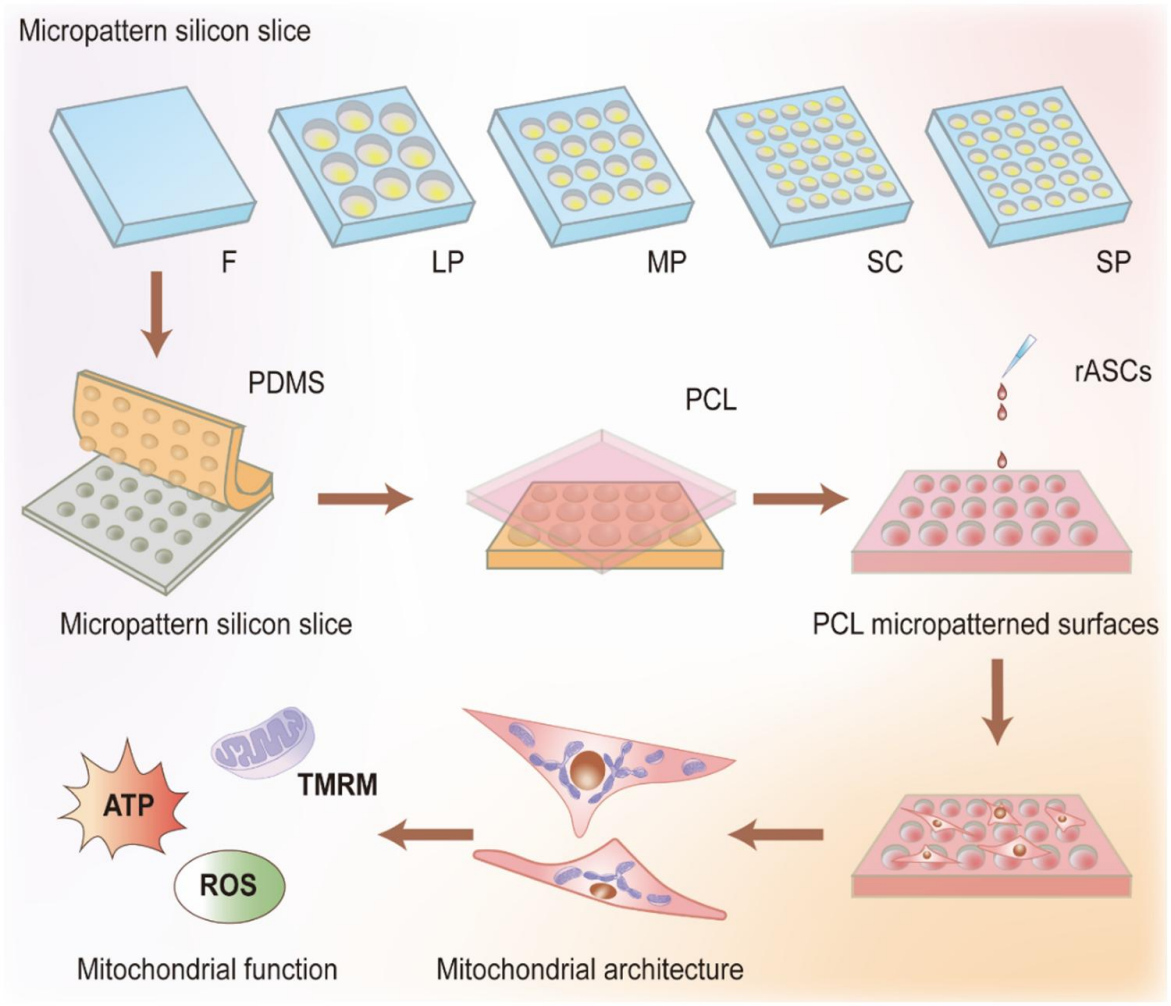
Table of contents. Schematic illustration of preparation process of PCL substrate with micropattern and its effect on mitochondrial structure and function of rat adipose-derived mesenchymal stem cells (rASCs).

Polycaprolactone (PCL) is one of the most commonly used Food and Drug Administration-approved polymers because of its tunable biodegradability and excellent biocompatibility. Currently, it has been utilized as the raw material of novel absorbable medical devices [30, 31]. In this study, a PCL substrate with micropatterns was used as a model to study the influence of surface microtopology on mitochondrial architecture and function in rat adipose-derived mesenchymal stem cells (rASCs) (Figure 1). Specifically, rASCs were cultured on five kinds of micropatterned PCL films, including plane (F), large pit (LP) with a diameter of 100 μm, medium pit (MP) with a diameter of 30 μm, small pit (SP) with a diameter of 5 μm and small column (SC) with a diameter of 5 μm, which were obtained by the melt casting method after laser etching. The mitochondrial architecture in cells on micropatterned faces was examined and analyzed. Mitochondrial function, including the mitochondrial membrane potential, the release of reactive oxygen species (ROS) and ATP production, was evaluated.

## Materials and methods

### Preparation and characterization of micropatterned PCL surfaces

Micropatterned PCL surfaces were fabricated using photolithography and casting. In simple terms, photolithography was used to prepare silicon wafer molds with five different micropatterned surfaces, including flat (F), LP (diameter = 100 μm; depth = 20 μm; spacing = 50 μm), MP (diameter = 30 μm; depth =6 μm; spacing =30 μm), SP (diameter = 5 μm; depth =6 μm; spacing = 5 μm) and SC (diameter = 5 μm; height = 6 μm; spacing = 5 μm). Next, a mixture of a polydimethylsiloxane (PDMS) base and curing agent (10:1 w/w) (Sylgard, USA) was cast on the master silicon wafer and cured at 90°C for 1 h to obtain the PDMS master wafer. Weigh 60 g PCL particles and dissolve in 500 ml dichloromethane, stir until dissolved (overnight), measure 50 ml of the dissolved substance and pour it into an iron disk (the disk is covered with the dissolved substance), and put it in a fume hood to evaporate until film formation. Then, using the PDMS master as a template, a replica of PCL (molecular weight = 80 000, Sigma-Aldrich, USA) with a designed micropattern was obtained by casting (80°C, 2 h). Flat PCL surfaces were prepared using a smooth PDMS template. All substrate surfaces were pretreated with the complete medium (CM) for 24 h before seeding cells. To characterize the micropatterns on the PCL surfaces, field emission scanning electron microscopy (Merlin, Carl Zeiss, Germany) was used. The micropatterned surfaces used in this study are listed in Supplementary Table S1.

### Cell culture

rASCs (p4-p6, Cyagen Biosciences, China) were propagated in α-MEM (Gibco, USA) supplemented with 10% fetal bovine serum (FBS, Gibco) and 1% penicillin-streptomycin (Gibco). rASCs were routinely subcultured at 80% confluence. Unless otherwise specified, the seeding density of cells was 30 000 cells cm^−2^.

### Crystal violet staining

After being fixed with 4% paraformaldehyde, the samples were incubated with a crystal violet solution (Solarbio, China) for 15 min. The staining was observed with a stereomicroscope (Nikon, Japan).

### Immunofluorescence

For fixed cell staining, after being fixed with 4% paraformaldehyde, the stain was washed after incubation and the staining was observed with a confocal laser scanning microscope (Zeiss, Germany). The FAK was stained with Anti-FAK (phospho Y397) antibody [EP2160Y] (ab81298) and Goat Anti-Rabbit IgG H&L (Alexa Fluor^®^ 647) (ab150079). The cytoskeletal F-actin was stained with 488-labeled phalloidin (ACROBiosystems, China). The nucleus in cells were labeled by DAPI (Beyotime Biotechnology, China).

### Live cell staining

The mitochondria in rASCs were labeled by incubating rASC cultures with 200 nM MitoTracker Green (Beyotime Biotechnology, China) at 37°C for 20 min. The mitochondrial membrane potential was examined by incubating rASC cultures with 200 nM TMRM (AAT Bioquest, USA) at 37°C for 30 min. For ROS detection, the samples were incubated using an ROS assay kit (Beyotime Biotechnology) at 37°C for 30 min. After staining, the staining was observed with a confocal laser scanning microscope (Zeiss).

### Image analysis

ImageJ/Fiji (with the MiNA plugin) was used for all image analyses [32]. For analysis, the image is first converted to binary by thresholding, where a foreground pixel is assigned the maximum value (255) and background pixels are assigned the minimum possible value (0) [32]. After mitochondria were restored by image processing, objects consisting of only 1 or 0 branches were defined as individual mitochondrial particles, of which 0 branches were a point. Objects that consist of multiple branches are called mitochondrial networks. The number and length of mitochondrial particles and networks were counted to characterize the mitochondrial architecture in each cell. Mitochondria were divided into individual mitochondrial particles and mitochondrial networks when analyzing the structure of mitochondrial networks. Objects composed of only one or zero branches are called individual mitochondrial particles. Mitochondrial networks are composed of multiple branches. The length of each mitochondrial rod and each branch of the mitochondrial network is referred to as the mitochondrial length. The number of branches that make up a mitochondrial network is known as the complexity of that mitochondrial network.

### Intracellular ATP content

The ATP levels of the samples were determined using the ATP Assay Kit (Beyotime Biotechnology) according to the manufacturer’s instructions. Briefly, the medium was replaced with ATP stabilizer lysis reagent (200 μl/sample), and cell lysis solution was added in 100 μl duplicates to a black microplate. Luminescence was read on a luminometer (Tecan, Switzerland). The ATP content was corrected for the background reading and standard substance. ATP levels are expressed as nmol/mg of proteins.

### Quantitative real-time polymerase chain reaction

Total RNA from different groups was extracted using a micro RNA extraction kit (Magen, China, R4114) according to the manufacturer’s instructions. After obtaining total RNA, a reverse transcription kit (TaKaRa, Japan, RR047A) was used to reverse-transcribe RNA into cDNA. Afterward, quantitative real-time PCR was performed on a LightCycler 96 system (Nest, China) using All-In-one qPCR Mix (GeneCopoeia, QP001, USA). The fold change of each target gene was normalized to the housekeeping gene glyceraldehyde-3-phosphate dehydrogenase (GAPDH) and calculated using the 2^−ΔΔCt^ method. The sequences of primers used in this study are listed in Supplementary Table S2.

### Statistical analysis

To ensure repeatability, independent tests were carried out three times with a minimum of triplicates for each group. GraphPad Prism 9 software (GraphPad) was used for statistical analysis. All data are expressed as the mean ± standard deviation (SD). Statistical significance was determined using one-way analysis of variance (ANOVA) followed by Tukey’s test. A value of *P* < 0.05 was considered statistically significant.

## Results

### Effects of micropatterns on cell spreading

**Figure 2.**
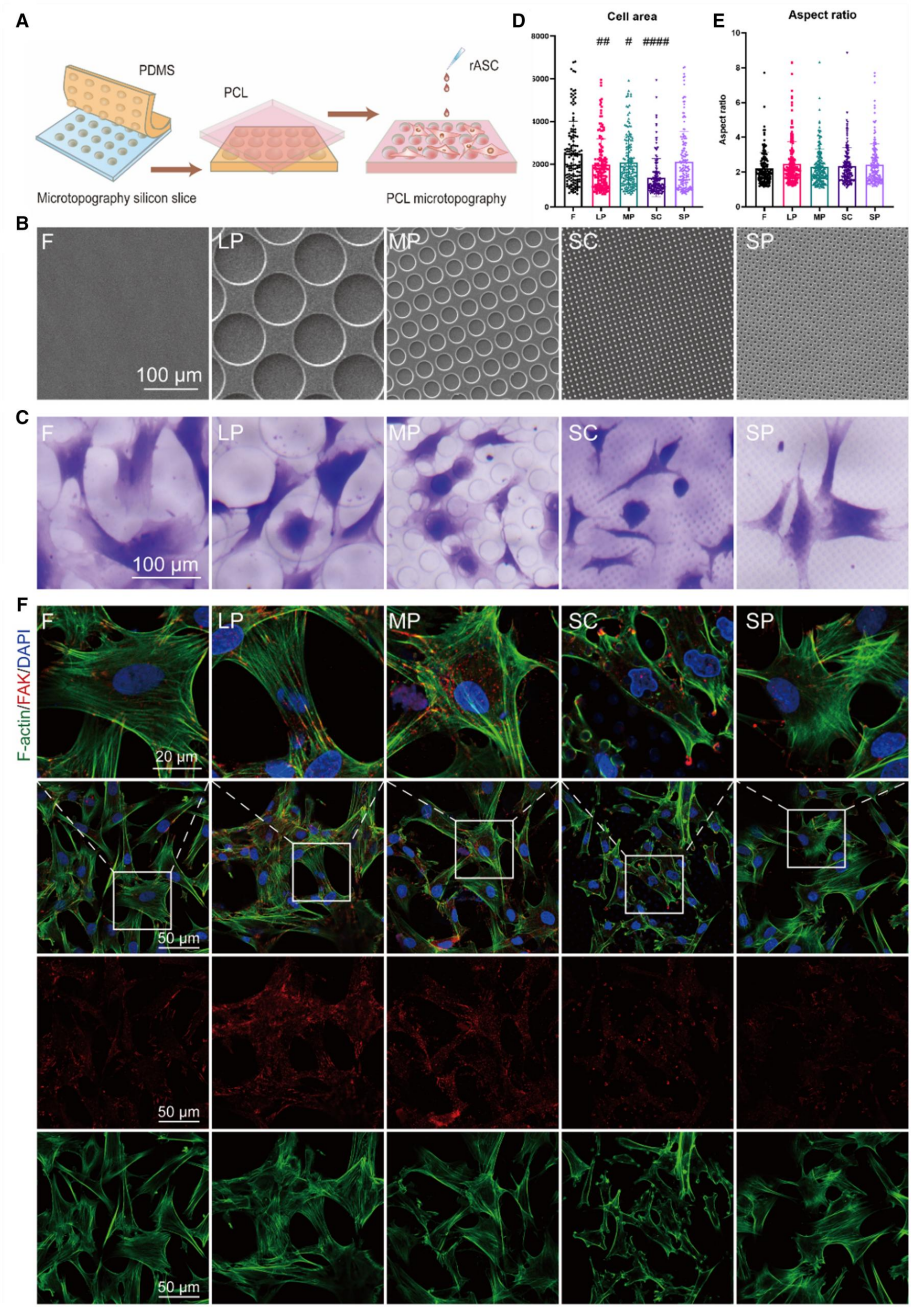
Cell culture on different micropatterned surfaces. (**A**) Experimental design. (**B**) SEM images of micropatterned surfaces. (**C**) Representative images of cell morphology characterized by crystal violet staining. (**D**) Cell area statistics. (**E**) Cell aspect ratio statistics. (**F**) Representative images of cell FAK and F-actin characterized by immunofluorescence (#*P* < 0.05, ##*P* < 0.01, ###*P* < 0.001, ####*P* < 0.001 vs. *F*).

We first prepared and characterized the surface of the micropatterned surfaces and statistically analyzed the cell spread on micropatterned surfaces. As shown in Figure 2B, five different micropatterned surfaces, including flat (F), LP, MP, SP and SC, were fabricated successfully in good accordance with the design.

The hydrophilic/hydrophobic properties of the micropatterned surfaces were determined by measuring the water contact angle. Although the water contact angles of LP and MP were greater compared to F under dry conditions, all surfaces became relatively hydrophilic after being pretreated with the complete medium (CM), with no significant difference in the water contact angle compared to F (Supplementary Figure S1). Since all substrate surfaces were pretreated with CM for 24 h before seeding cells, we believe the potential observed differences in mitochondria networks and functions can be attributed to surface pattern-induced mechanical stimuli rather than surface chemistry.

Next, we evaluated the spreading state of rASCs on various surfaces. Apparently, rASCs were spread well on F and SP (Figure 2). There was no significant change in the rASC cell area on SP compared with F. In contrast, the cell area on LP, MP and SC was reduced compared to F. Among them, the cell area of SC decreased the most, while the cell area of LP and MP was slightly limited. No significant difference in the cell aspect ratio among all micropatterned surfaces was observed.

We performed the staining for focal adhesion kinase (FAK) and cytoskeletal F-actin (Figure 2F). The results showed that FAK in all groups was arranged aligned to actin fibers, many of them appear at the end of stressed fibers formed by F-actin. The expression of FAK was higher in LP and MP groups, and the intracellular distribution of F-actin was more dense. The cells in the SC group formed actin rings around the microcolumn and large FAK plaques mainly located on the actin rings. Accordingly, the nuclei of some cells in SC appear to be pushed away and deformed, which is consistent with previous studies[33].

### Effects of micropatterns on mitochondrial architecture in rASCs

To clarify the changes in mitochondrial network architecture in rASCs on the micropatterned surfaces, we first used MitoTracker Green to label the mitochondria in rASCs. The mitochondria of each cell on the micropatterned surfaces were then analyzed statistically after the mitochondrial network structure was restored by ImageJ/Fiji (with the MiNA plugin) [32].

**Figure 3.**
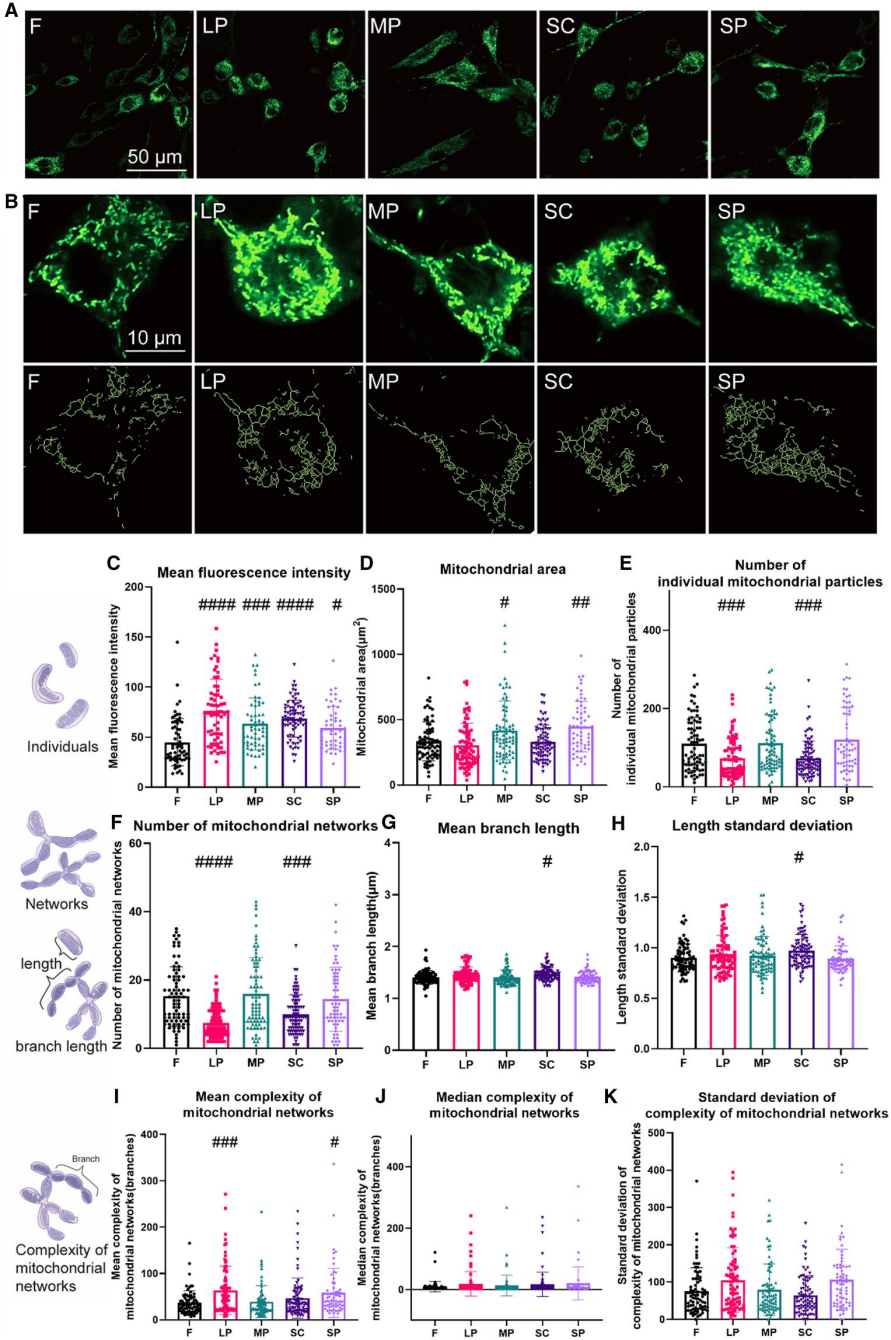
The mitochondrial network architecture in rASCs on micropatterned surfaces. (**A**) Representative images of mitochondria stained with MitoTracker green dye. (**B**) Representative images of mitochondria in a single rASC dyed with MitoTracker green dye (line 1) and mitochondrial morphological structures recovered after image processing (line 2). For analysis, the image is first converted to binary by a threshold value, and then the branches of the mitochondria are calculated, with zero or one mitochondrial branch as an individual mitochondrial particle and two or more mitochondrial branches as a mitochondrial network. (**C**) Mean fluorescence intensity of MitoTracker green staining. (**D**) The total area of all mitochondria in each cell. (**E**) Total number of individual mitochondrial particles (point mitochondria with zero branches and chondriokont with one branch) in each cell. (**F**) Total number of mitochondrial networks in each cell. Mitochondria containing two or more branches are recorded as mitochondrial networks. (**G**) The average length of all mitochondrial branches in each cell. The length of all individual mitochondrial particles and the length of each branch in the mitochondrial networks were measured to obtain the average length of mitochondrial branches for each cell. (**H**) The standard deviation of mitochondrial branch lengths in each cell, reflecting the degree of dispersion of all mitochondrial branch lengths in each cell. (**I**) The average complexity of the mitochondrial network in each cell. The complexity of the mitochondrial network was characterized by calculating the number of branches contained in each mitochondrial network. (**J**) The median complexity of the mitochondrial network in each cell. The median branch numbers of the mitochondrial network in each cell. (**K**) The standard deviation of the complexity of the mitochondrial network in each cell, reflecting the dispersion degree of the branch numbers of the mitochondrial network in each cell (#*P* < 0.05, ##*P* < 0.01, ###*P* < 0.001, ####*P* < 0.001 vs. *F*).

As shown in Figure 3A, the mitochondria in most cells were distributed throughout the cytoplasm. The average fluorescence intensity of MitoTracker Green in cells on each micropatterned surface was significantly upregulated compared with that in cells on F (Figure 3C). In addition, we calculated the area of mitochondria on the micropatterned surfaces. Compared with the mitochondria in cells on LP, SC and F, the area of mitochondria in MP and SP was significantly larger (Figure 3D). The three-dimensional distribution of mitochondria in rASCs on micropatterned surfaces was also examined. It seemed that various micropatterns did not affect the three-dimensional distribution of mitochondria in cells, as mitochondria in most cells on all surfaces were distributed throughout the cytoplasm (Supplementary Figure S2).

Next, we analyzed the morphological structure of the mitochondrial networks. In Figure 3B, the first row shows representative images of mitochondria in cells on micropatterned surfaces, while the second row shows the mitochondrial morphological structure restored after processing the images of the first row using ImageJ/Fiji (with the MiNA plugin) [32]. Apparently, cells on all micropatterned surfaces formed a certain degree of interconnected mitochondrial networks. Mitochondria were then divided into individual mitochondrial particles and mitochondrial networks for statistical purposes [32]. First, we focused on the total number of individual mitochondrial particles contained in each cell. Among all groups, the total number of individual mitochondrial particles in each cell in the LP and SC groups was significantly reduced (Figure 3E). Furthermore, we evaluated the number and complexity of mitochondrial networks. The total number of mitochondrial networks in each cell in the LP and SC groups was significantly reduced (Figure 3F). And the mean branch length of mitochondria in SC increased (Figure 3G). We also evaluated the size distribution of mitochondrial branches. The fraction of short mitochondrial branches (<1 μm) in rASCs on LP, SC and SP decreased, and the fraction of long branches (>1 μm) increased (Supplementary Figure S3), indicating that these surfaces tended to induce long mitochondrial branches. The complexity of mitochondrial networks on micropatterned surfaces can be characterized by counting the number of branches. What’s more, the mitochondrial networks in the LP and SP groups contained more branches (Figure 3I). There was no significant difference in the median and standard deviation of the number of branches contained in the mitochondrial networks on different micropatterned surfaces (Figure 3J and K).

### Gene expression related to mitochondrial dynamics in rASCs on micropatterned surfaces

**Figure 4.**
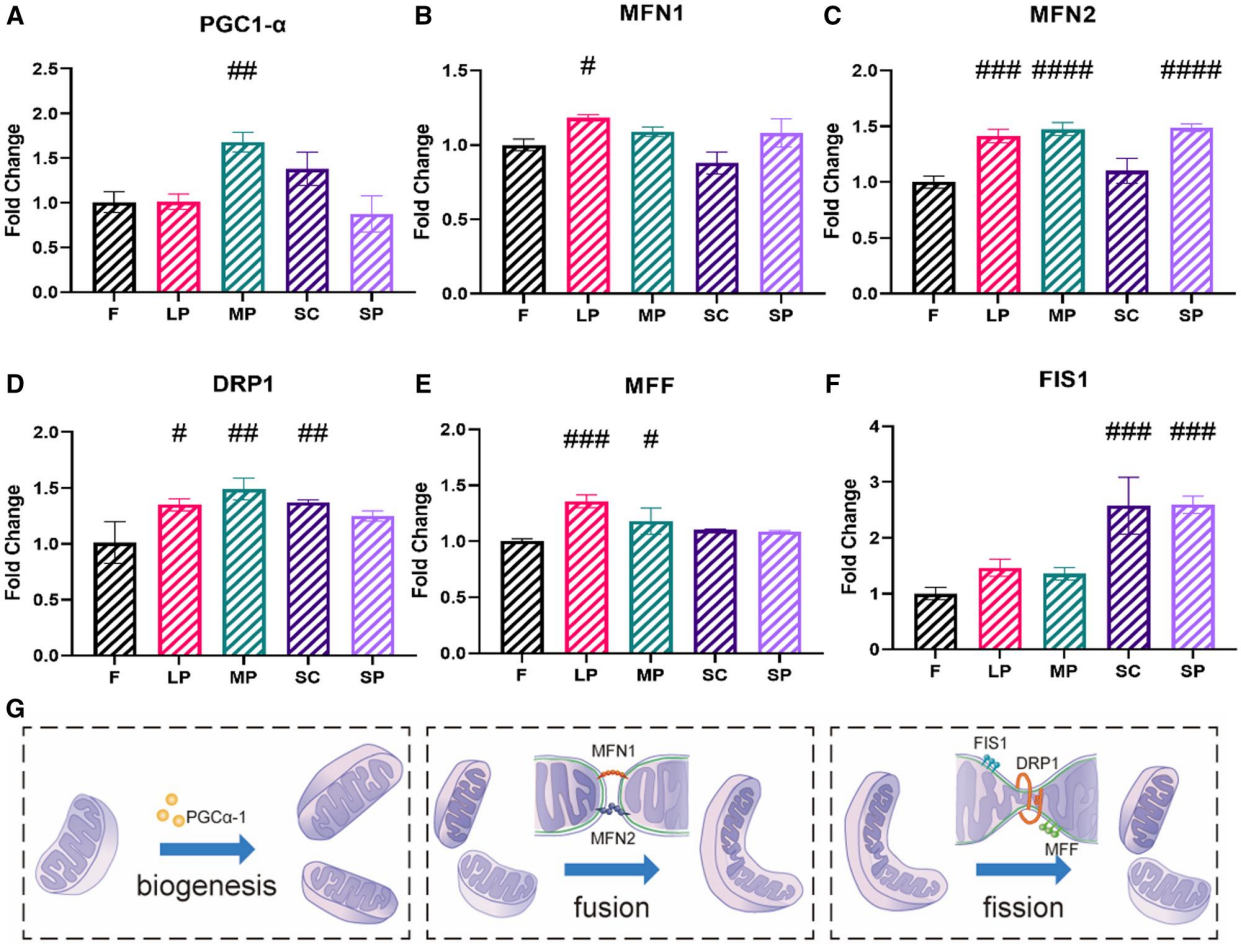
The expression of genes related to mitochondrial dynamics in rASCs on micropatterned surfaces. (**A**) The expression of peroxisome proliferator-activated receptor γ-coactivator 1-α (PGC1-α). (**B**) The expression of mitofusin-1 (MFN1). (**C**) Expression of mitofusin-2 (MFN2). (**D**) Expression of mitochondrial dynamin-related protein 1 (DRP1). (**E**) The expression of mitochondrial fission factor (MFF). (**F**) The expression of mitochondrial fission protein 1 (FIS1). (**G**) The structural regulation of mitochondria by the above genes is divided into mitochondrial biogenesis (PGC1-α, left), mitochondrial fusion (MFN1, MFN2, middle), and mitochondrial fission (DRP1, MFF, FIS1, right) (#*P* < 0.05, ##*P* < 0.01, ###*P* < 0.001, ####*P* < 0.001 vs. *F*).

As Figure 4G, recent studies have identified a series of key genes that control mitochondrial dynamics[3]. Peroxisome proliferator-activated receptor gamma-coactivator 1α (PGC1-α) is a transcriptional coactivator that is a key inducer of mitochondrial biogenesis in cells. Quantitative analysis of PGC1-α mRNA levels showed that MP increased the expression of PGC1-α in rASCs (Figure 4A). The dynamic mitochondrial network architecture is regulated by two opposing processes, mitochondrial fission and fusion. Both mitofusin-1 (MFN1) and mitofusin-2 (MFN2) are critical for mitochondrial fusion [34], while mitochondrial fission is mainly regulated by dynamin-related protein 1 (DRP1), mitochondrial fission factor (MFF) and mitochondrial fission protein 1 (FIS1) [35]. As expected, the expression of MFN1 and MFN2 in rASCs on the LP was upregulated (Figure 4B and C). Moreover, MP and SP upregulated MFN2 expression (Figure 4C).

As shown in Figure 4D–F, the expression of DRP1 and MFF in rASCs on LP and MP was upregulated, indicating stimulated mitochondrial midzone fission. Meanwhile, higher mRNA levels of DRP1 and FIS1 in rASCs on SC were observed, suggesting elevated peripheral fission (Figure 4E).

### Effects of micropatterns on mitochondrial functions in rASCs

**Figure 5.**
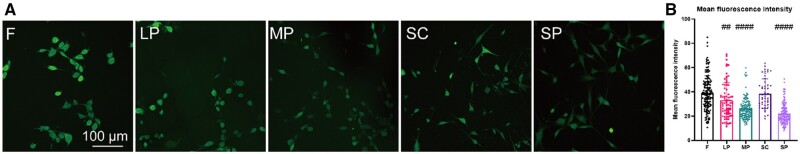
Production of ROS by rASCs on micropatterned surfaces. (**A**) Representative images of ROS localized in rASCs stained with DCFH-DA. (**B**) Mean fluorescence intensity of ROS (#*P* < 0.05, ##*P* < 0.01, ###*P* < 0.001, ####*P* < 0.001 vs. *F*).

Mitochondria are the main source of intracellular ROS because mitochondrial oxidative phosphorylation (OXPHOS) produces large amounts of ROS as byproducts [16]. Here, we also determined the intracellular ROS in rASCs on micropatterned surfaces. All micropatterned surfaces induced lower ROS production than F, except for SC (Figure 5A and B).

**Figure 6.**
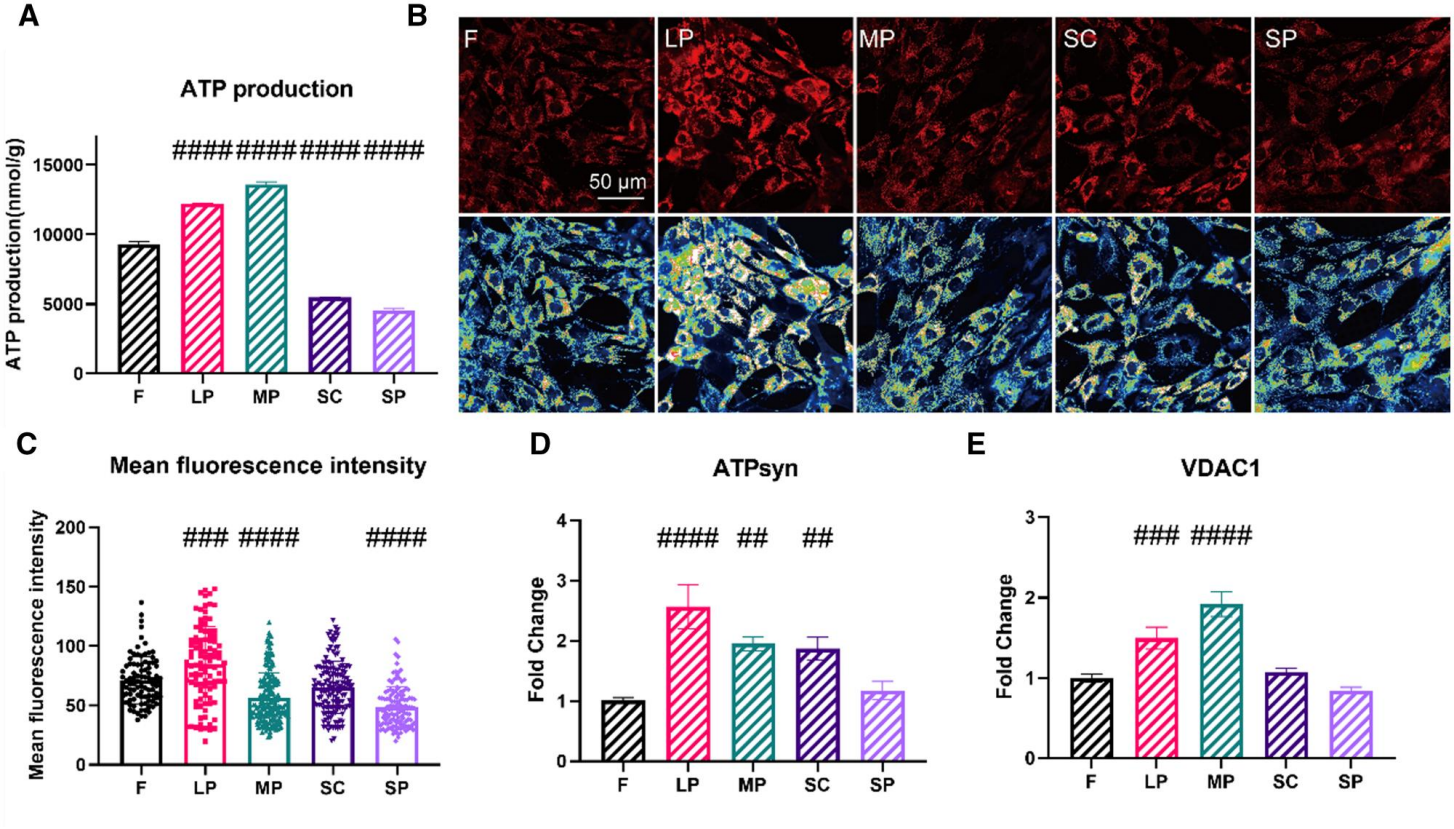
Mitochondrial function related to ATP production. (**A**) Intracellular ATP levels. (**B**) Representative images of mitochondrial membrane potential in rASCs stained with TMRM (line 1). Representative images of the heatmaps of mitochondrial membrane potential in rASCs (line 2). (**C**) Mean fluorescence intensity of TMRM. (**D**) The expression of ATPsyn. (**E**) The expression of VDAC-1 (#*P* < 0.05, ##*P* < 0.01, ###*P* < 0.001, ####*P* < 0.001 vs. *F*).

One major function of mitochondria is to produce ATP. Thus, the ATP level was examined (Figure 6A). According to our results, the intracellular ATP in rASCs on LP and MP was significantly increased. Meanwhile, it was decreased in rASCs on SC and SP compared with F. Next, we investigated the possible mechanism underlying the regulatory effects of micropatterned surfaces on ATP production in rASCs. The mitochondrial membrane potential (ΔΨm) generated by proton pumps (complexes I, III, IV) forms the transmembrane potential of hydrogen ions, which is harnessed to make ATP during OXPHOS. ΔΨm in rASCs on micropatterned surfaces was determined via TMRM staining. As shown in Figure 6B and C, the ΔΨm in the LP group was the highest, while that in the MP and SP groups was the lowest. There was no significant difference between SC and F. We then detected the mRNA levels of mitochondrial ATP synthase (ATPsyn) and voltage-dependent anion channel 1 (VDAC1). The expression of ATPsyn was markedly higher in the LP, MP and SC groups, with LP having the highest expression (Figure 6D). Meanwhile, LP and MP also stimulated the expression of VDAC1 (Figure 6E).

## Discussion

Nowadays, in the application and mechanism exploration of regenerative biomaterials, the interaction between cells and biomaterials is emphasized [36]. Considering that the microscopic morphology of the substrate can change the cell morphology and internal cytoskeleton, and affect cell differentiation and paracrine functions, we hypothesized that the microtopological surface can regulate mitochondria [28, 29]. In this study, PCL substrates with micropatterns were used as models to investigate the effect of surface microtopology on mitochondrial structure and function of rat adipose mesenchymal stem cells.

In our previous studies, we found that soft substrates induced MSCs to secrete more prorepair factors and exhibit less spread morphology with a diameter of 10–30 μm [37]. It seems that the cell spreading state is a key factor that affects MSC functions. Thus, we designed micropatterns that can induce full-cell spreading (LP) and partial cell spreading (MP) (Figure 2B). We also designed SC with columns much smaller than cells to restrict spreading. SP was used as the mirror pattern of SC.

In this study, we focused on clarifying the regulatory effects of micropatterned surfaces on mitochondria. All patterned surfaces were made of PCL to exclude the potential effects of substrate materials. Besides, the hydrophilic/hydrophobic properties of the micropatterned surfaces were determined by measuring the water contact angle in the resubmission (Supplementary Figure S1) to exclude the potential effects of surface chemistry. Thus the observed differences in mitochondria networks and functions can be attributed to the surface pattern induced mechanical stimuli.

Next, the spreading state of rASCs on various surfaces were evaluated (Figure 2C). The results indicated that the micropatterned surfaces can regulate the area of rASCs, and the pore structure has no significant effect on the cell aspect ratio. Furthermore, SC, LP and MP all significantly reduced the cell area, and the cell area on SC was significantly lower than that on LP and MP.

We performed the staining for FAK and cytoskeletal F-actin (Figure 2F). Mitochondria tightly associate with the cytoskeleton within cells, allowing them to sense mechanical stimuli originating from both intracellular and environmental sources conveyed by the cytoskeleton, and to respond accordingly [20, 38]. Besides, studies have shown that FAK signaling regulates mitochondrial morphology and function in cardiomyocytes [39]. Therefore, these results suggest that micropatterned surfaces may affect mitochondrial network architecture and function through regulating cytoskeleton and focal adhesion.

The mitochondrial network architecture in rASCs on micropatterned surfaces were evaluated. It seemed that various micropatterns did not affect the distribution of mitochondria in cells, as mitochondria in most cells on all surfaces were distributed throughout the cytoplasm (Figure 3A and Supplementary Figure S2). Then, the total number of mitochondrial networks in each cell was tested. Considering that the number of mitochondrial individuals and networks in the LP and SC groups were significantly reduced (Figure 3E and F), while the mitochondrial area of the two groups was not less than that of the other groups (Figure 3D), mitochondria in LP and SC may form larger networks. In addition, mitochondrial networks in SC have longer branches (Figure 3G). According to our results, the mitochondrial networks in the LP and SP groups contained more branches, indicating that LP and SP induced mitochondria to form more complex networks (Figure 3I). It has been shown that different cell spreading states affect mitochondrial architecture[40, 41]; thus, the observed different mitochondrial architecture in cells may be attributed to the spreading state of cells induced by different micropatterned surfaces.

Quantitative analysis of PGC1-α mRNA levels showed that MP increased the expression of PGC1-α in rASCs (Figure 4A), which may further lead to stimulated biogenesis of mitochondria. Indeed, the mitochondrial area in cells on MP was larger than that on F, LP and SC (Figure 3D). Previous studies have demonstrated that PGC1-α is highly responsive to environmental cues, such as temperature and nutritional status [42]. Our results showed that microtopographic cues also affected the expression of Pgc1-α. Interestingly, the level of PGC1-α mRNA was not changed in SP, although it also induced a larger mitochondrial area. Thus, SP may increase mitochondrial biogenesis through other pathways.

As expected, the expression of MFN1 and MFN2 in rASCs on the LP was upregulated (Figure 4B and C), which was consistent with the larger and more complex mitochondrial network observed in rASCs on the LP (Figure 3). MP-upregulated MFN2 expression but did not increase mitochondrial fusion, possibly due to the synergistic action of many proteins in mitochondrial fusion. There was no significant change in the mitochondrial outer membrane fusion protein level of SC compared with that of F (Figure 4B and C), which also indicated that although the number of mitochondria in SC was reduced (Figure 3E and F), a large mitochondrial network was not necessarily formed, but a longer mitochondrial network was formed. SP upregulated MFN2 expression and formed a complex mitochondrial network (Figure 4C).

Mitochondrial fission is divided into midzone fission and peripheral fission, which are quite different in function and mechanism. DRP1 can trigger both types, while MFF governs only endoplasmic reticulum- and actin-mediated mitochondrial midzone fission [30]. In contrast, FIS1, which is located in the outer membrane of mitochondria, regulates only peripheral fission. According to our results, the expression of DRP1 and MFF in rASCs on LP and MP was upregulated, indicating stimulated mitochondrial midzone fission (Figure 4D and E). Generally, fission at the midzone leads to mitochondrial proliferation [30]. However, the number of individual mitochondria in rASCs on LP and MP was not increased compared with that of other groups. Considering that the expression of MFF was also increased in LP and MP, the coordination of multiple fission and fusion regulators may help rASCs reach a homeostatic state that is optimal for cellular energy metabolism. When the mitochondrial fission site is at the periphery of the organelle, the resulting daughter mitochondrion is soon cleared by mitophagy [30]. This may explain the decreased number of individual mitochondria particles in rASCs on SC (Figure 4E).

Mitochondria are the main source of intracellular ROS, but excessive generation of ROS is usually associated with harm and indicates impaired mitochondrial functions [32, 43]. All micropatterned surfaces induced lower ROS production than F, except for SC (Figure 5A and B). Therefore, the level of ROS in the micropatterned faces is normal and will not cause damage to cells.

Indeed, one major function of mitochondria is to produce ATP. In this study, we review the key factors related to ATP production and discuss the mechanisms by which micropatterned surfaces regulate ATP production. It has been reported that mitochondria exhibit higher ATP production capacity when they fuse into large networks, which is consistent with the results of LP [13] (Figures 3E, F and I and 4A). For the MP, the increased ATP in rASCs may be attributed to enhanced mitochondrial biogenesis (Figures 3D and 4A). ATPsyn is essential for ATP production since it is the enzyme that catalyzes the synthesis of ATP driven by an influx of protons across the inner membrane of mitochondria [32]. After ATP is produced, it is transported to the cytoplasm to fuel cellular energy. Voltage-dependent anion channel 1 (VDAC1), a major component of the mitochondrial outer membrane, mediates ATP translocation across the outer membrane of mitochondria [25]. Thus, the elevated intracellular ATP levels in rASCs on LP may be a result of the promoted OXPHOS process, ATPsyn expression and VDAC1 expression. In contrast, the increased level of ATP in the MP group may be mainly attributed to ATPsyn and VDAC1, rather than OXPHOS. The decreased ΔΨm in rASCs on SP may cause the corresponding lower ATP production (Figure 6A and C). Interestingly, it was noted that the expression of both ATPsyn and VDAC1 was increased in rASCs on SC (Figure 6D), but ATP production dropped (Figure 6A). One possible explanation is that the relatively lower ΔΨm in rASCs on SCs drives ATPsyn to produce ATP less efficiently (Figure 6B and C).

The importance of mitochondria is self-evident, and the method of regulating mitochondria has gradually become a part of the design of biomaterials. As the mechanical sensitivity of mitochondria is well known, the mechanical regulation of mitochondria by biomaterial surface design has come into public view. Our study illustrates the regulation of micropatterned surfaces for mitochondria, highlighting the potential of LP and MP as simple platforms for stimulating mitochondrial and subsequent mesenchymal stem cell function.

## Conclusion

**Figure 7.**
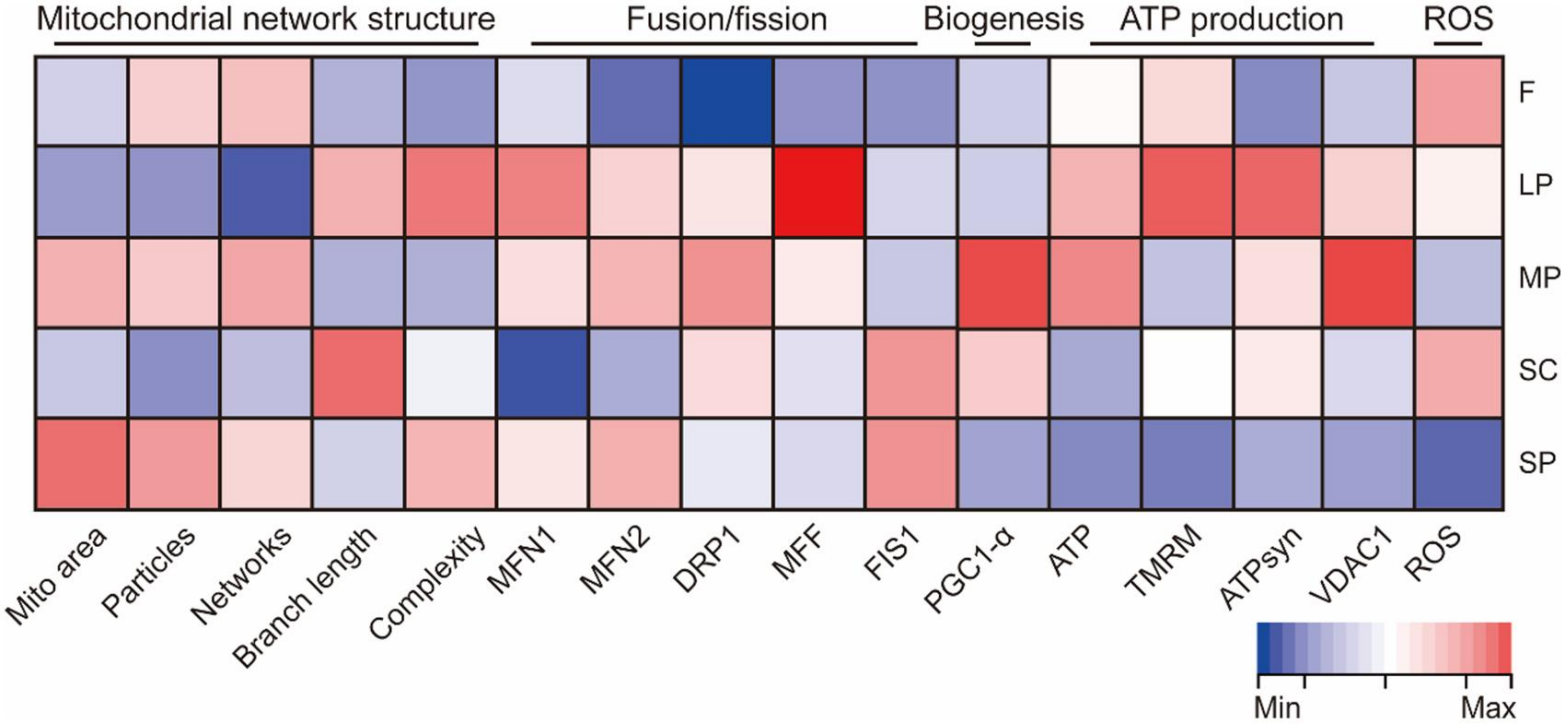
Summary of changes in mitochondrial network structures and functions by micropatterned surfaces. All mitochondria-related representations were normalized using the Sangerbox Bioanalysis tool and demonstrated with a heatmap.

The present study demonstrated that different micropatterns (LP, MP, SP and SC) affected the mitochondrial network architecture and functions in rASCs. As shown in Figure 7, both LP and MP promoted ATP production, although through different mechanisms. LP stimulated rASCs to form larger and more complex mitochondrial networks, enhanced the OXPHOS process and stimulated ATPsyn and VDAC1 expression. Meanwhile, the elevated ATP in MP may mainly be attributed to enhanced mitochondrial biogenesis and VDAC1 expression. Our results revealed the significance of micropatterns in regulating mitochondria.

## Supplementary Material

rbae052_Supplementary_Data
