# Supplementary material for: Regulation of mitochondrial network architecture and function in mesenchymal stem cells by micropatterned surfaces
Source: Regen Biomater. 2024 May 7;11:rbae052. doi: 10.1093/rb/rbae052 (PMC11162196; doi:10.1093/rb/rbae052)
Supplement: rbae052_Supplementary_Data [file rbae052_supplementary_data.docx]

**Supporting Information**

Regulation of mitochondrial network architecture and function in mesenchymal stem cells by micropatterned surfaces

Zixuan Dong^1,3,4^, Weiju Han^2,3,4^, Panyu Jiang^1,3,4^, Lijing Hao^2,3,4,*^, Xiaoling Fu^3,4,*^

1. The Second Affiliated Hospital, School of Biomedical Sciences and Engineering, South China University of Technology, Guangzhou 511442, P. R. China

2. School of Materials Science and Engineering, South China University of Technology, Guangzhou 510006, P. R. China.

3. National Engineering Research Center for Tissue Restoration and Reconstruction and Innovation Center for Tissue Restoration and Reconstruction, Guangzhou 510006, P. R. China.

4. Laboratory of Biomedical Engineering of Guangdong Province, South China University of Technology, Guangzhou 510006, P. R. China.

*. Correspondence address. School of Materials Science and Engineering, South China University of Technology, Guangzhou 510006, P. R. China. Email:  [msljhao@scut.edu.cn](mailto:msxlfu@scut.edu.cn).

*. Correspondence address. School of Biomedical Sciences and Engineering, South China University of Technology, Guangzhou International Campus, Guangzhou 511442, P. R. China. Email: [msxlfu@scut.edu.cn](mailto:msxlfu@scut.edu.cn).

## Supplementary Figures


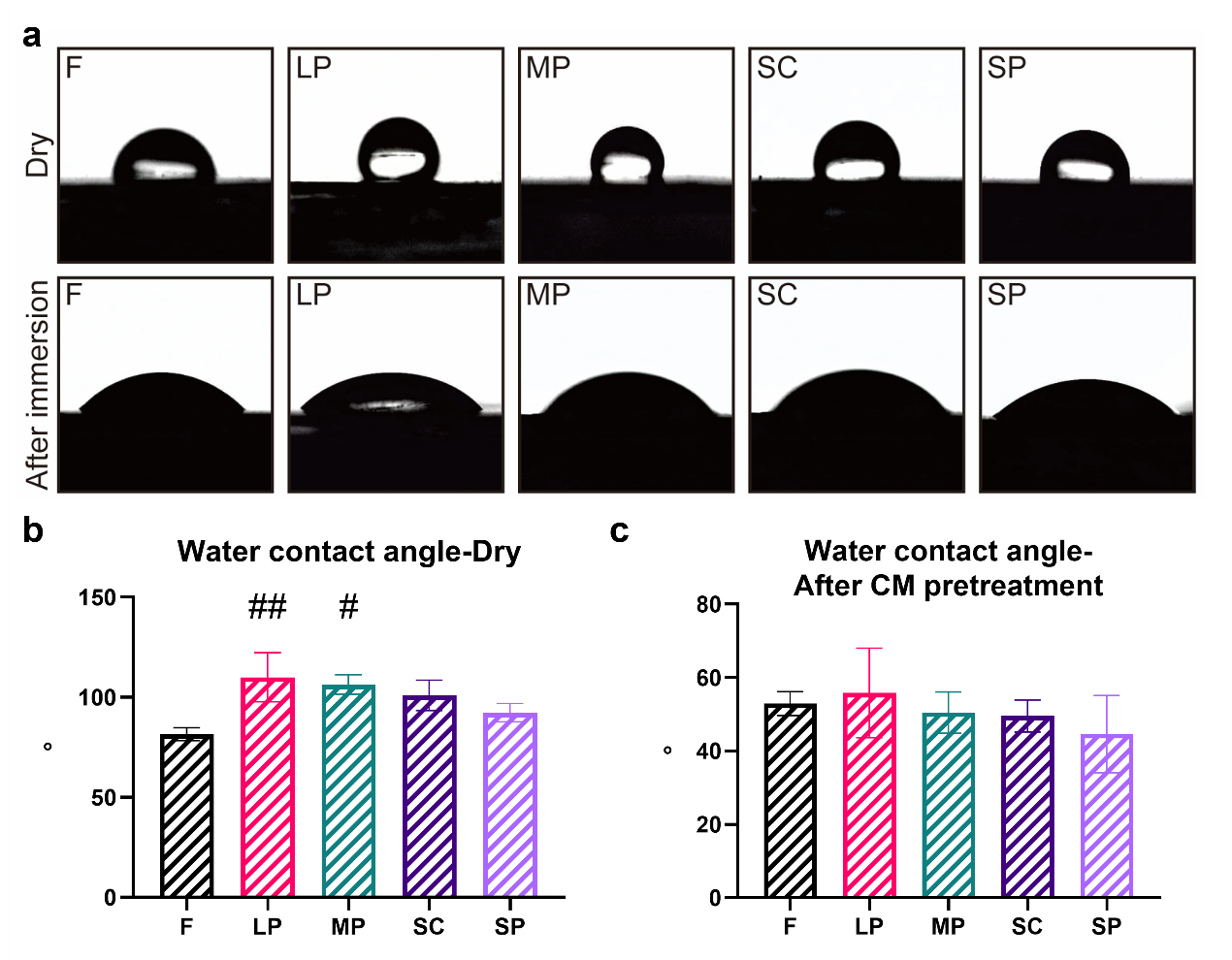


Figure S1. The water contact angle of various micropatterned surfaces. a. Representative photographs illustrating the contact angle for water drops deposited onto dry micropatterned surfaces (Row 1) and onto micropatterned surfaces after pretreatment in the cell culture medium （CM） (Row 2). b-c. Statistical analysis of the water contact angle of dry micropatterned surfaces (b) and micropatterned surfaces after CM pretreatment (c). (#p < 0.05, ##p < 0.01, ###p < 0.001, ####p < 0.001 vs F).


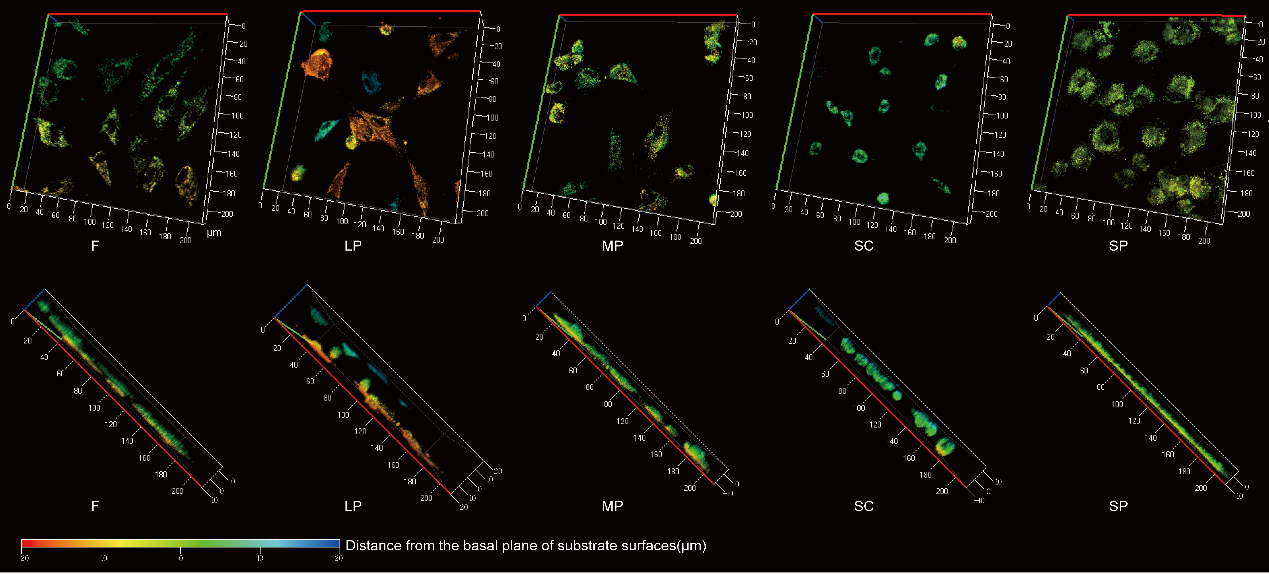


Figure S2. Spatial distribution of mitochondrial network structures in rASC on micropatterned surfaces. The differences in color of the heat map indicated the distance between the mitochondria and the basal plane of substrate surfaces.


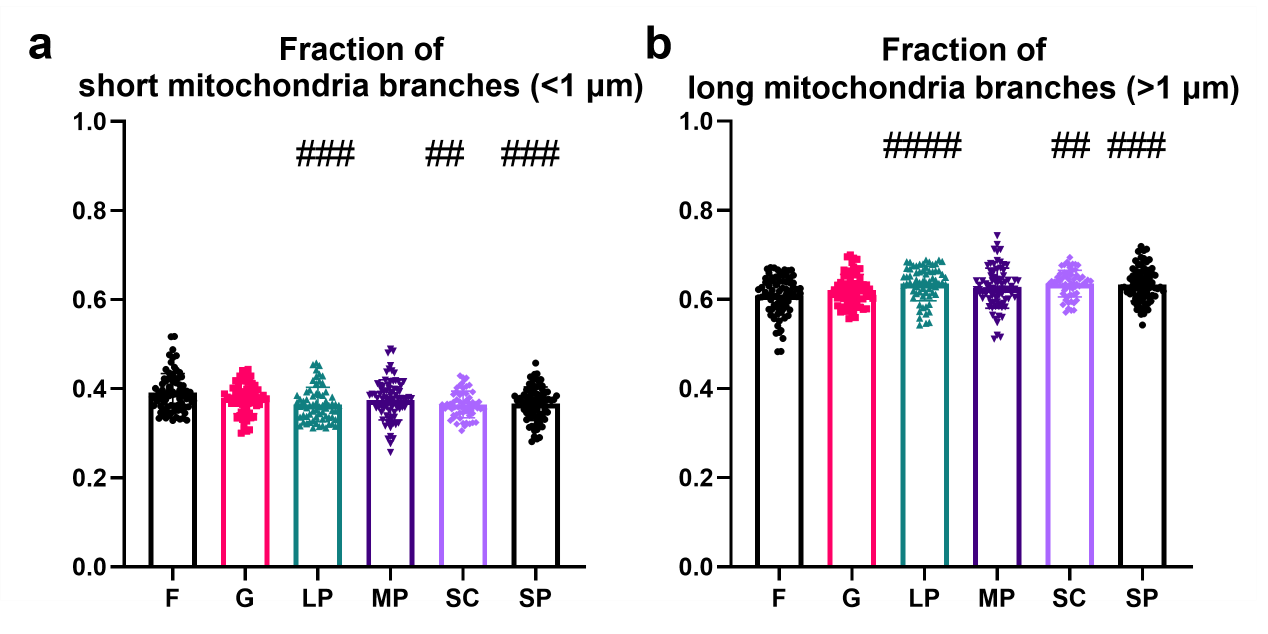


Figure S3. Size distribution of mitochondrial branches. a. Fraction of short mitochondrial branches (<1 µm). Ratio of short mitochondrial branches to all mitochondrial branches in each cells. b. Fraction of long mitochondrial branches (>1 µm). Ratio of long mitochondrial branches to all mitochondrial branes in each cells. (#p < 0.05, ##p < 0.01, ###p < 0.001, ####p < 0.001 vs F).

## Materials and methods

## Preparation and characterization of micropatterned PCL surfaces

The micropatterned surfaces used in this study are listed in Table 1.

| micropatterned surfaces | Diameter(μm) | depth/height(μm) | spacing(μm) |
| --- | --- | --- | --- |
| LP | 100 | 20 | 50 |
| MP | 30 | 6 | 30 |
| SC | 5 | 6 | 5 |
| SP | 5 | 6 | 5 |

Supplementary Table S1. Characters of micropatterned surfaces

## Water contact angle

5 μL droplets of water were deposited onto micropatterned surfaces using a standard automated goniometer (DSA25). And the resulting image of the liquid–air interface analyzed with ImageJ/Fiji (with the Contect Angle plugin). All angles were averaged over at least three measurements on different areas of a micropatterned surfaces.

## Quantitative real-time polymerase chain reaction

The primer sequences used in this study are listed in Table S1.

| Rat Genes | Forward Primer 5’→3’ | Reverse Primer 5’→3’ |
| --- | --- | --- |
| GADPH | ATGGTGGTGAAGACGCCAGTA | GGCACAGTCAAGGCTGAGAATG |
| VDAC1 | ATGTGAATGATGGGACGGAGTTTGG | CGACCTGATACTTGGCTGCTATTCC |
| ATPsyn | GGTAGCGTTGGTATATGGGCAGATG | CCTGGGTGAAGCGGAAGATGTTG |
| PGC1-α | CCACTACAGACACCGCACACATC | GTATTCGTCCCTCTTGAGCCTTTCG |
| MFN1 | GTGGGCTGGAAACTCATCTCTGTC | CAAACTGCTGCTTAAACGCTCTCTC |
| MFN2 | TCCACAGCCATTGCCAGTTCAC | CCGCACAGACACAGGAAGAAGG |
| DRP1 | ACAGCGTCCCAAAGGCAGTAATG | CCATGTCCTCGGATTCAGTCAGAAG |
| MFF | CTCTGGCGCTGAAAACACCT | ACTGCTCGGATCTCTTCGCT |
| FIS1 | GAATACGCCTGGTGCCTGGTTC | GAAGACATAATCCCGCTGCTCCTC |

Supplementary Table S2. Primer sequences used for qRT‒PCR
